# Clinical Analysis of Relapse Risk in Immune-Checkpoint-Inhibitor-Related Pneumonitis

**DOI:** 10.3390/jcm15072481

**Published:** 2026-03-24

**Authors:** Kanae Maruyama, Mitsuhiro Abe, Takeshi Kawasaki, Dai Horiuchi, Noriko Sakuma, Shinsuke Kitahara, Daisuke Ishii, Izumi Ohno, Yuichi Takiguchi, Takuji Suzuki

**Affiliations:** 1 Department of Respirology, Graduate School of Medicine, Chiba University, 1-8-1 Inohana, Chuo-ku, Chiba-shi 260-8670, Chiba, Japan; 2 Department of Respirology, Japanese Red Cross Narita Hospital, 90-1, Iida-cho, Narita-shi 286-8523, Chiba, Japan; 3 Department of Respirology, Funabashi Central Hospital, 6-13-10 Kaijin, Funabashi-shi 273-8556, Chiba, Japan; 4 Department of Respirology, Nissan Tamagawa Hospital, 4-8-1, Seda, Setagaya-ku 158-0095, Tokyo, Japan; 5 Department of Respirology, Chiba Rosai Hospital, 2-16, Tatsumidaihigashi, Ichihara-shi 290-0003, Chiba, Japan; 6 Department of Medical Oncology, Graduate School of Medicine, Chiba University, 1-8-1 Inohana, Chuo-ku, Chiba-shi 260-8670, Chiba, Japan; 7 Department of Medical Oncology, Sannou Hospital, 166-2, Sannou-cho, Inage-ku, Chiba-shi 263-0002, Chiba, Japan

**Keywords:** immune checkpoint inhibitors (ICIs), checkpoint inhibitor-related pneumonitis (CIP), relapse, steroid therapy, lung injury

## Abstract

**Background**: While immune checkpoint inhibitor (ICI)-related pneumonitis (CIP) may relapse during or after steroid treatment, clinical factors associated with CIP relapse are unclear. This study explored risk factors potentially associated with CIP relapse. **Methods**: This single-center retrospective study included 1099 patients who received ICIs at our institution between April 2015 and March 2022. Among them, 39 patients who developed CIP and were treated with systemic steroids and tapered to prednisolone (PSL) ≤ 20 mg/day were analyzed. Patients were classified into relapse and non-relapse groups based on whether CIP recurred during or after steroid treatment. Patient characteristics, clinical features at onset, and treatment strategies were compared between the two groups. **Results**: Thirteen patients (33.3%) experienced relapse. Compared with the non-relapse group, the relapse group had a significantly higher proportion of non-smokers (30.8 vs. 3.3%, *p* = 0.035), a greater frequency of Common Terminology Criteria for Adverse Events (CTCAE) Grade 2 pneumonitis (92.3 vs. 53.8%, *p* = 0.029), and lower serum KL-6 levels (288 vs. 704 U/mL, *p* = 0.014). The relapse group also had a shorter duration of steroid therapy at the initial dose, ≥0.5 mg/kg/day, ≥15 mg/day, and ≥20 mg/day (*p* < 0.05) and lower cumulative steroid doses (1140 vs. 1902 mg, *p* = 0.015). Relapse tended to occur in patients with relatively mild pneumonitis who received lower steroid doses and shorter treatment durations. **Conclusions**: Non-smoking status, CTCAE Grade 2 pneumonitis, lower serum KL-6 levels, shorter duration of steroid therapy, and lower cumulative steroid dose were potentially associated with CIP relapse. Adequate steroid dosing and tapering may help prevent relapse.

## 1. Introduction

Immune checkpoint inhibitors (ICIs) are human monoclonal antibodies that enhance T-cell-mediated antitumor immune responses. They selectively target receptors expressed on the surfaces of T cells or tumor cells that contribute to immune evasion, such as cytotoxic T-lymphocyte-associated protein-4 (CTLA-4), programmed cell death 1 (PD-1), and programmed cell death ligand 1 (PD-L1). By blocking these pathways, ICIs restore antigen-presenting capacity to helper T cells and enhance the cytotoxic function of activated T cells [1,2].

ICIs have improved treatment outcomes in advanced malignancies, and their use has increased substantially. Consequently, the management of immune-related adverse events (irAEs) has become increasingly important [3]. IrAEs can affect multiple organs, inducing hepatitis, colitis, myocarditis, dermatitis, hypophysitis, hypothyroidism, and adrenal insufficiency [3,4]. Among these, checkpoint-inhibitor-related pneumonitis (CIP) requires prompt treatment at onset, as it can be fatal due to respiratory failure [5].

Pneumonitis associated with anticancer therapies, including CIP, is graded based on the Common Terminology Criteria for Adverse Events ver5.0 (CTCAE v5.0). According to the CTCAE criteria, Grade 1 pneumonitis is defined as asymptomatic radiographic findings only; Grade 2 includes symptomatic cases with clinical features such as fever, cough, or dyspnea that limit activities of daily living; and Grade 3 or higher represents severe disease requiring oxygen supplementation [6]. The reported incidence of CIP varies depending on cancer type and the specific agent used, ranging from 0 to 8.3% for all grades according to CTCAE and from 0 to 4% for CTCAE Grade 3 or higher [7,8]. While CIP most commonly develops within 2–3 months of initiation of ICI therapy, it can occur any time, including after the first administration or following prolonged treatment exceeding one year [7,9].

Previously reported risk factors for CIP include those traditionally associated with drug-induced lung injury, such as male sex, advanced age, smoking history, pre-existing lung diseases, including interstitial lung abnormalities, prior thoracic radiotherapy, lung cancer, and combination therapy with other agents, such as cytotoxic chemotherapeutic drugs [5,10,11,12,13,14,15,16,17,18,19]. Organizing pneumonia is the most frequently reported radiologic pattern in CIP, accounting for approximately 23–65% of cases; however, diffuse alveolar damage has also been observed in severe cases [7,20,21].

Although the precise mechanisms underlying the development of CIP remain incompletely understood, excessive activation and proliferation of T cells with subsequent infiltration into pulmonary tissue are thought to play a central role. This process is further promoted by increased secretion of inflammatory cytokines and impaired regulatory T cell function, ultimately resulting in lung injury [22,23].

The National Comprehensive Cancer Network (NCCN) Clinical Practice Guidelines in Oncology recommend discontinuation of ICIs and initiation of corticosteroid therapy for CTCAE Grade 2 or higher pneumonitis [24]. Steroid therapy for CIP is generally effective. However, relapse during steroid tapering has been reported [25,26,27]. Sata et al. found that 3.8% of patients with non-small cell lung cancer treated with nivolumab experienced CIP relapse. They further noted that an organizing pneumonia (OP) pattern on imaging at onset and reduction in PSL to less than 5 mg/day were characteristic of relapse cases [27]. During CIP treatment, anticancer therapy must be suspended, and relapse-associated prolongation of CIP management may have significant consequences, including delays in resuming treatment for the primary malignancy.

Although several risk factors for CIP have been identified [5,10,11,12,13,14,15,16,17,18,19], few studies have comprehensively investigated CIP relapse in relation to patient characteristics, cancer types, ICI regimens, and corticosteroid treatment strategies.

The aim of this study was to explore clinical features associated with CIP relapse to identify potential treatment-related considerations to prevent relapse. The null hypothesis was that there are no differences in patient characteristics or steroid treatment parameters between patients with and without CIP relapse.

## 2. Materials and Methods

### 2.1. Ethics

This single-center retrospective study was conducted in accordance with the amended Declaration of Helsinki. The research protocol was approved by the Human Ethics Committee of Chiba University Hospital (approval number: 2265). Written informed consent was obtained from all patients, or consent was waived through an opt-out procedure.

### 2.2. Patients

We retrospectively reviewed the medical records of 1099 consecutive patients who received ICIs, including nivolumab, pembrolizumab, atezolizumab, durvalumab, avelumab, and ipilimumab, at Chiba University Hospital between April 2015 and March 2022. Among these patients, 72 (6.5%) were diagnosed with CIP.

The treating physician diagnosed CIP based on the appearance of new abnormal chest findings on CT within 12 months after the last administration of ICIs, together with clinical, radiological, and laboratory information, after excluding alternative diagnoses such as infection, heart failure, or cancer progression [28]. Infectious pneumonia was excluded based on clinical symptoms; laboratory findings, such as procalcitonin, β-D-glucan, and mycoplasma pneumoniae antibodies; and response to antimicrobial therapy. Cardiogenic pulmonary edema and other causes of pulmonary congestion were evaluated based on clinical findings and imaging studies. Tumor progression, including lymphangitic carcinomatosis or metastatic disease, was excluded through radiological assessment and clinical course.

**Figure 1:**
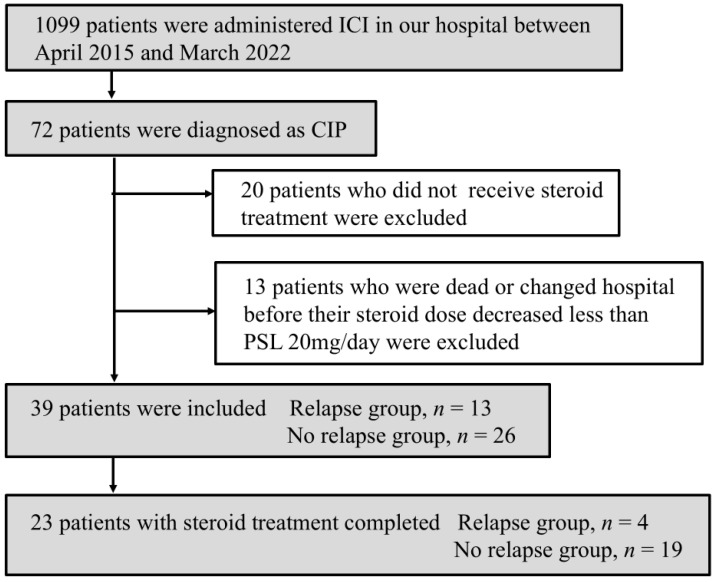
Flowchart of patient selection. Among patients treated with immune checkpoint inhibitors, those diagnosed with CIP and treated with systemic steroids were included in the analysis. ICIs, immune checkpoint inhibitors; CIP, checkpoint-inhibitor-related pneumonitis; PSL, prednisolone.

Patients who did not receive corticosteroid therapy (*n* = 20) and those who died or were transferred to another hospital before the PSL dose was tapered to <20 mg/day (*n* = 13) were excluded. Consequently, 39 patients (3.5%) were included in the final analysis. The primary treatment strategy in each case was discontinuation of ICIs and systemic corticosteroid therapy, with the initial dose and tapering schedule determined by the treating physician based on disease severity and clinical response. Among the 23 patients who completed corticosteroid therapy, we similarly classified them into relapse and non-relapse groups and evaluated differences in the duration of PSL administration at doses below the moderate range (Figure 1).

Patients were classified into two groups: those who experienced relapse during or after corticosteroid treatment and those who did not relapse after steroid discontinuation or dose reduction to <10 mg/day of PSL as maintenance therapy. Relapsed CIP was defined as radiologic exacerbation on X-ray or CT following initial improvement with corticosteroid therapy during steroid tapering or after discontinuation, with other potential causes excluded. The corticosteroid dose and tapering schedule were determined at the discretion of the treating clinician.

We compared patient characteristics, pattern of CIP onset, and treatment strategies between the relapse and non-relapse groups.

### 2.3. Clinical Data Collection

We collected and analyzed data from medical records, including patient background, pattern of onset, and treatment-related factors. Patient background variables comprised age, sex, smoking history, primary cancer type, history of thoracic radiotherapy, presence of lung abnormalities on CT prior to initiation of ICIs, sites of primary and metastatic disease, interstitial lung abnormalities, emphysema, old inflammatory changes, serum creatinine level, estimated glomerular filtration rate (eGFR), antinuclear antibody status, type of ICI, number of ICI administrations, concomitant medications, and the interval from first ICI dose to the onset of CIP. Pattern of CIP onset included CTCAE grade; serum levels of lactate dehydrogenase (LDH), C-reactive protein (CRP), and Krebs von den Lungen-6 (KL-6); and radiologic findings on chest CT. Treatment-related variables included the total duration of corticosteroid therapy; the number of days of PSL at the initial dose, ≥0.5 mg/kg/day, ≥15 mg/day, and ≥20 mg/day; the cumulative corticosteroid dose; and the initial steroid dose.

CT findings at onset were independently reviewed and classified by two pulmonologists (KM and SK) into six radiologic patterns according to the American Thoracic Society (ATS) and the European Respiratory Society classification of idiopathic interstitial pneumonias [29,30]: (1) OP, (2) non-specific interstitial pneumonia (NSIP), (3) DAD, (4) hypersensitivity pneumonitis, (5) bronchiolitis, and (6) other or unclassifiable patterns.

### 2.4. Statistical Analysis

The normality of continuous variables was assessed using the Shapiro–Wilk test. Because the sample size was small and most variables, except for the estimated glomerular filtration rate (eGFR, mL/min/1.73 m^2^) and the Brinkman index, were not normally distributed, continuous variables are presented as medians and interquartile ranges (IQRs). Comparisons between groups were performed using the Mann–Whitney U test. Categorical variables were compared using Fisher’s exact test and are presented as percentages. A two-sided *p*-value < 0.05 was considered statistically significant.

False discovery rate (FDR)-adjusted *Q*-values were calculated as a supplementary measure to account for multiple comparisons; however, given the exploratory nature of this study, primary interpretation was based on unadjusted *p*-values.

Receiver operating characteristic (ROC) curves were constructed for continuous variables with statistically significant differences to determine optimal cut-off values for predicting CIP relapse. These variables were subsequently dichotomized according to the identified cut-off values, and we performed univariate and multivariate analyses with CIP relapse as the dependent variable.

All statistical analyses were conducted using EZR software version 1.55 (Saitama Medical Center, Jichi Medical University, Saitama, Japan) [31].

## 3. Results

### 3.1. Patient Backgrounds

The characteristics of the 39 patients are summarized in Table 1. The median age was 72 years (IQR 67–77). Most patients were male (*n* = 35, 90%) and had a history of smoking (*n* = 34, 89%). Lung cancer was the most common primary malignancy (*n* = 28, 71%). Pembrolizumab was the most frequently used ICI (*n* = 23, 59%), and the most common CT pattern at onset was OP (*n* = 18, 46%). The mean duration of steroid therapy was 101 ± 52 days. Twenty-three patients (59%) received a starting PSL dose of 0.5 mg/kg/day.

Among the 39 participants, 13 (33.3%) experienced relapse. The characteristics of patients in the relapse group are shown in Table 2**.** The median number of days from the first ICI dose to relapse was 231 (IQR 128–318); the median from the last dose to relapse was 94 days (IQR 80–156); and the median from initiation of steroid therapy to relapse was 74 days (IQR 59–140). The median PSL dose at relapse was 5.0 (IQR 0–7.5) mg/day, and nine of the 13 patients relapsed while receiving a PSL dose of less than 5 mg/day.

### 3.2. Cancer Treatment Progress After CIP

**Figure 2:**
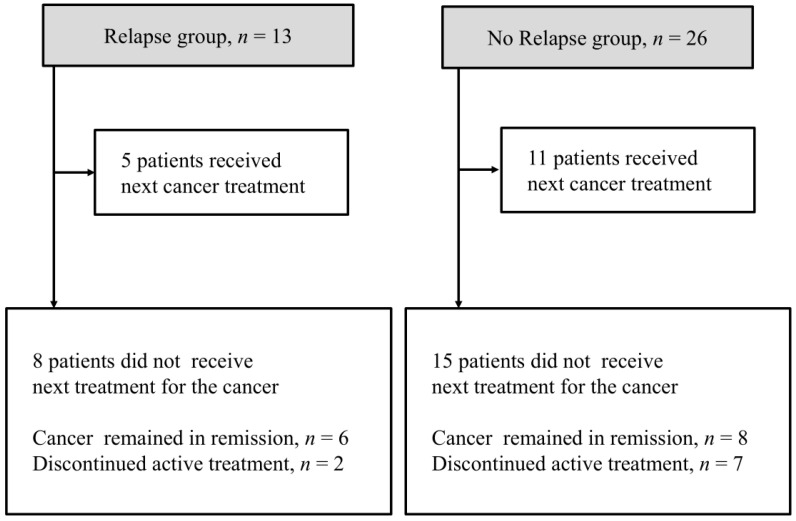
Cancer treatment progress after CIP. Clinical course after steroid treatment in patients with immune-checkpoint-inhibitor-related pneumonitis (CIP). CIP, checkpoint-inhibitor-related pneumonitis; PS, performance status.

Figure 2 shows the course of cancer treatment in the 39 patients after the onset of CIP. Sixteen patients (44.4%) were able to resume treatment for their primary malignancy after CIP; five of these patients belonged to the relapse group. No patients underwent ICI rechallenge because all patients in this study had CIP of CTCAE Grade 2 or more.

Of the 23 patients who did not receive subsequent-line treatment, 10 discontinued active treatment due to the unavailability of alternative agents or a deterioration in performance status during CIP treatment. The remaining 13 patients were managed with observation alone because the primary disease remained in remission.

### 3.3. Risk Factors for Relapse

**Figure 3:**
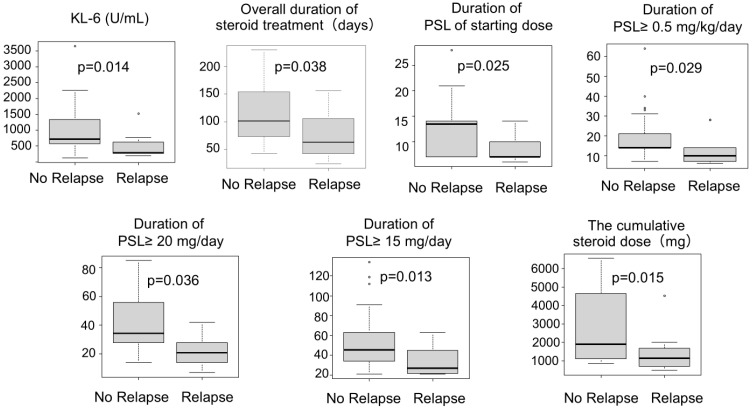
Box plot of continuous variables showing significant differences in the Mann–Whitney U test. Serum KL-6 levels at the onset of CIP were significantly lower in the relapse group. The duration of steroid therapy was also significantly shorter in the relapse group, and the cumulative PSL dose was significantly lower in the relapse group (*p* = 0.015). CIP, checkpoint-inhibitor-related pneumonitis; PSL, prednisolone.

Table 1 summarizes the statistical comparisons between the relapse and no-relapse groups, and Figure 3 presents box plots of continuous variables with significant differences. The relapse group included a significantly higher proportion of patients without a history of smoking than the no-relapse group (4/13 [30.8%] vs. 1/26 [3.3%], *p* = 0.035). CTCAE Grade 2 pneumonitis was significantly more frequent in the relapse group (12/13 [92.3%] vs. 14/26 [53.8%], *p* = 0.029), and serum KL-6 levels at onset were significantly lower (288 (IQR 268–625) vs. 704 (IQR 538–1248) *p* = 0.014). CT findings at the onset of CIP did not differ significantly between the two groups; however, an OP pattern was observed in 46% (*n* = 6/13) of patients in the relapse group, whereas all five patients with a DAD pattern belonged to the no-relapse group.

The duration of steroid therapy was significantly shorter in the relapse group. Overall duration from initiation of steroid therapy to completion or maintenance dose was shorter (63 (IQR 42–106) vs. 101 (IQR 74–150), *p* = 0.038), as were the duration of the initial steroid dose (7 (IQR 7–10) vs. 14 (IQR 8–14), *p* = 0.025) and the number of days with PSL ≥ 0.5 mg/kg/day (10 (IQR 7–14) vs. 14 (IQR 14–21), *p* = 0.029), PSL ≥ 20 mg/day (21 (IQR 14–28) vs. 35 (IQR 28–56), P=0.0036), and PSL ≥ 15 mg/day (27 (IQR 22–45) vs. 46 (IQR 34–63), *p* = 0.013). The cumulative PSL dose was also significantly lower in the relapse group (1140 (IQR 700–1688) vs. 1902 mg (IQR 1155–4531), *p* = 0.015). There was no significant difference in the starting PSL dose between groups.

**Figure 4:**
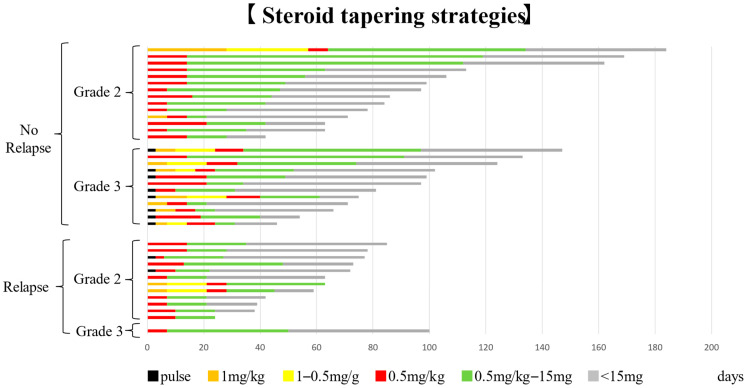
Steroid tapering strategies in the 39 patients. Initial PSL dose tended to be lower in patients with Grade 2 pneumonitis and higher in those with Grade 3 pneumonitis. The duration of steroid therapy, particularly the duration of high- to medium-dose treatment, tended to be shorter in the relapse group. PSL, prednisolone.

Figure 4 illustrates individual steroid treatment courses and tapering strategies according to CIP severity. In the relapse group, 69% of patients (9/13) were started on PSL at 0.5 mg/kg/day. Among all patients with CTCAE Grade 2 CIP, 77% (20/26) were started on PSL 0.5 mg/kg/day, and this proportion was 66% (8/12) in the relapse group.

An additional analysis was performed across the 23 patients who completed steroid treatment (Table 3), including 4 patients in the relapse group and 19 in the no-relapse group. In this subset, the number of days with PSL ≥ 0.5 mg/kg/day (7 (IQR 7–9) vs. 14 (IQR 14–21), *p* = 0.031), ≥15 mg/day (21 (IQR 21–23) vs. 42 (IQR 35–58), *p* = 0.0056), and ≥20 mg/day (21 (IQR 21–23) vs. 42 (IQR 35–58), *p* = 0.0064) was significantly lower in the relapse group. No significant differences were observed in the duration of treatment with lower-dose PSL (<15 or <20 mg/day).

**Figure 5:**
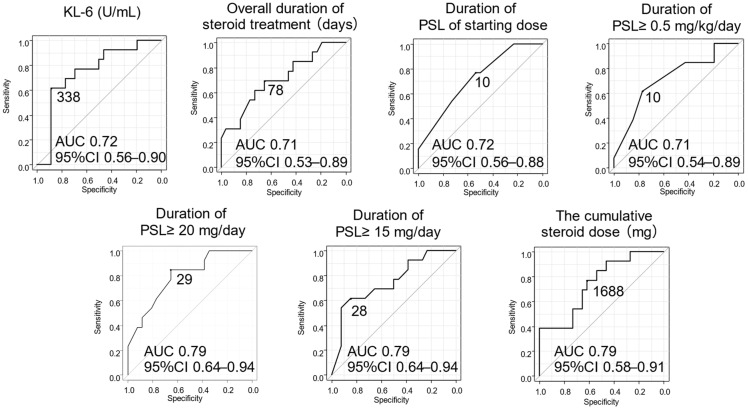
Receiver operating characteristic (ROC) curves for continuous variables showing significant differences. ROC, receiver operating characteristic; AUC, area under the curve; PSL, prednisolone.

ROC curves were used to determine cut-off values for continuous variables that showed significant differences in predicting CIP relapse (Figure 5). These variables were subsequently binarized, and univariate analysis was performed with CIP relapse as the outcome (Table 4). The univariate analysis identified the following as significant risk factors for relapse: no smoking history (*p* = 0.042); CTCAE Grade 2 (*p* = 0.036); KL-6 at onset ≤ 338 U/mL (*p* = 0.0028); overall time from steroid initiation to completion or maintenance dose ≤ 78 (*p* = 0.047); duration of PSL 0.5 mg/kg/day or more ≤10 days (*p* = 0.023), PSL 15 mg/day or more ≤28 days (*p* = 0.0058), and PSL ≥ 20 mg/day ≤ 29 days (*p* = 0.0073); and cumulative PSL dose ≤ 1688 mg (*p* = 0.031).

A multivariate logistic regression analysis was performed with three variables (non-smoking status, CTCAE Grade 2 pneumonitis, and serum KL-6 ≤ 338 U/mL) significantly associated with relapse in the univariate analysis. In the multivariate model, serum KL-6 ≤ 338 U/mL remained significantly associated with CIP relapse (odds ratio [OR] 14.9, 95% confidence interval [CI] 2.14–104, *p* = 0.006). In contrast, non-smoking status (OR 9.63, 95% CI 0.69–134, *p* = 0.091) and CTCAE Grade 2 pneumonitis (OR 6.99, 95% CI 0.57–85.2, *p* = 0.127) were not statistically significant in the multivariate analysis (Table 5).

## 4. Discussion

In this retrospective, real-world study, non-smoking status, lower serum KL-6 levels at onset, and CTCAE Grade 2 or less were identified as potential risk factors for CIP relapse. Furthermore, lower cumulative steroid dose and shorter duration of steroid therapy were associated with CIP relapse. Notably, patient characteristics and onset patterns observed in the relapse group differed from conventional risk factors and poor prognostic indicators for drug-induced lung injury. This paradoxical finding suggests that pulmonary immune responsiveness may have been relatively preserved in patients who experienced relapse.

Smoking has been reported as a conventional risk factor for drug-induced lung injury, including CIP [10,14,18]. In contrast, a significantly higher proportion of patients in the relapse group were non-smokers in the present study. One possible explanation is suppression of local immune responses in smokers’ lungs. Okazaki reported that smoking impairs the antigen-presenting capacity of alveolar macrophages, reducing T cell activation and B-cell-mediated antibody production [32]. Other studies have shown that smoking decreases the phagocytic capacity of alveolar macrophages, impairs antigen recognition by T cells, and suppresses cytokine secretion, including IL-1 and IL-6, from alveolar macrophages [33,34,35,36]. In contrast, non-smokers may retain relatively preserved immune responsiveness, which could predispose them to immune reactivation and CIP relapse, given that immune activation is a key mechanism underlying CIP.

Serum KL-6 is a mucin-like glycoprotein expressed on type II alveolar epithelial cells that serves as a biomarker of alveolar epithelial injury and structural destruction. It correlates with disease activity in interstitial lung disease, including drug-induced lung injury [37,38], and higher levels have been reported in severe cases and patients who die compared to survivors [39]. However, in this study, lower serum KL-6 levels were statistically associated with CIP relapse in the multivariate analysis, although the wide confidence interval suggests considerable uncertainty, and the ROC-derived cut-off may be affected by overfitting in this small dataset because of a lack of external validation. Extensive alveolar epithelial damage and architectural destruction, which result in elevated KL-6 levels, are thought to impair the immune functions of alveolar macrophages and epithelial cells [40,41]. When alveolar structures are relatively preserved, immune effector functions may remain intact, potentially facilitating immune reactivation and subsequent CIP relapse.

CTCAE grade 2 pneumonitis is a relatively mild clinical condition that does not require supplemental oxygen. The higher proportion of Grade 2 or milder cases in the relapse group may be confounded by factors such as non-smoking status and low serum KL-6 levels. In fact, non-smoking status and CTCAE Grade 2 pneumonitis were not statistically significant in the multivariate analysis. In non-smokers, baseline respiratory function is generally preserved and pre-existing lung disease is less common, while low KL-6 levels reflect limited alveolar epithelial injury and minimal structural lung destruction [39].

We also found an association with radiologic findings. All five patients with a DAD pattern were in the non-relapse group and classified as CTCAE Grade 3, indicating severe pneumonitis. In contrast, among the 12 patients with CTCAE Grade 2 pneumonitis in the relapse group, 50% exhibited an OP pattern. Although no statistically significant differences were observed in radiologic findings, OP was more frequent in the relapse group. Previous studies have reported that OP is associated with immune-checkpoint-inhibitor-related pneumonitis relapse. For example, Sata et al. [27] and Karayama et al. [42] identified the OP pattern as a characteristic feature of recurrent cases. OP is characterized by localized intra-alveolar lymphocytic infiltration with relatively preserved lung architecture [20,21,43], whereas DAD is associated with extensive irreversible structural destruction and fibrosis [20,21]. These findings further support our hypothesis that preserved lung structure and immune responsiveness may predispose patients to relapse.

Features suggestive of mild cases may have also influenced treatment strategies. Corticosteroids suppress T cell activity through their immunosuppressive effects, particularly at high doses. However, if immune suppression is insufficient, immune reactivation may occur during steroid tapering. This may be partly explained by the prolonged persistence of ICIs, which maintain strong antibody binding to T cells after treatment discontinuation [44,45].

As shown in Figure 4, 58% of patients were treated with an initial steroid dose of 0.5 mg/kg/day, accounting for 75% of patients with CTCAE Grade 2 pneumonitis. In contrast, 53% of patients with CTCAE Grade 3 pneumonitis received steroid pulse therapy. Even among patients initiated on 0.5 mg/kg/day, those in the relapse group tended to have shorter durations of moderate- to high-dose corticosteroid therapy.

According to the NCCN guidelines, CTCAE Grade 2 pneumonitis should be treated with prednisolone at 1–2 mg/kg/day, and CTCAE Grade 3 or higher should be treated with pulse methylprednisolone therapy, followed by gradual tapering over 4–6 weeks after improvement to CTCAE Grade 1 [24]. However, in this study, the initial steroid dose was often lower than recommended, and tapering was frequently initiated once radiologic improvement was observed. This finding suggests that the apparent association between shorter steroid duration or lower cumulative dose and relapse may not represent independent risk factors, but rather reflect treatment-related confounding. In real-world clinical practice, clinicians may hesitate to prescribe high-dose steroids for patients who do not require supplemental oxygen and can be managed in the outpatient setting. Additionally, the desire to resume anticancer therapy may contribute to earlier tapering. This tendency in milder cases may have resulted in insufficient steroid therapy, potentially contributing to CIP relapse.

Furthermore, an additional analysis of 23 patients who completed steroid therapy demonstrated that the relapse group had significantly shorter durations of high- and medium-dose steroid use, whereas no significant differences were observed in the duration of low-dose PSL (<15 mg/day). These findings suggest that prolonged low-dose maintenance therapy alone may not be effective in preventing relapse, whereas ensuring an adequate duration of high- to medium-dose steroid therapy is critical for sufficient suppression of immune activation. Effective early immunosuppression may prevent CIP relapse and shorten the overall duration of steroid treatment.

This study has several limitations. First, this was a retrospective study conducted at a single institution with a relatively small sample size, which may limit the generalizability of the findings. (1) Because the number of variables evaluated was relatively large compared with the sample size, the false discovery rate (FDR) should be considered when interpreting the *p*-values. (2) The multivariate analysis may have resulted in unstable estimates due to the limited number of events relative to the number of variables. The wide confidence intervals observed in the multivariate analysis indicate substantial uncertainty in the estimated effects and may reflect model instability due to the limited sample size. (3) Because the ROC-derived cut-off values were obtained from a relatively small dataset without external validation, the possibility of overfitting cannot be excluded. These cut-off values should be interpreted cautiously and require validation in larger prospective studies. (4) Because of the observational study design and small sample size, causal relationships between steroid treatment parameters and CIP relapse cannot be established, and unmeasured confounding factors may have affected the results. Second, patient characteristics were biased, as most patients were older, were male, had lung cancer, and were treated with pembrolizumab. There may have been imbalances between the relapse and non-relapse groups, which could have influenced the results. Especially, the study population was heterogeneous with respect to underlying malignancies, although lung cancer was predominant, and stratified analyses according to cancer type were not feasible due to the limited sample size. Third, no deaths were observed during the evaluation period in this cohort; therefore, competing risks regression with death as a competing event could not be performed. In larger cohorts where mortality events occur, competing risks analysis may be a more appropriate method for evaluating relapse outcomes. Finally, CIP diagnosis was based on clinical and radiological findings after exclusion of alternative diagnoses, and pathological confirmation was not available in all cases. Therefore, the findings of this study should be considered exploratory and hypothesis-generating. Further large-scale prospective studies are warranted to validate these observations.

Despite these limitations, this study has several strengths. We conducted a detailed evaluation of patient characteristics and steroid treatment courses, including treatment duration, dose intensity, and tapering patterns. Our findings suggest that relapse may occur relatively frequently even in patients with mild CIP, which has not been sufficiently emphasized in previous reports. Therefore, these findings may provide useful information for clinicians when determining appropriate steroid treatment schedules in clinical practice.

## 5. Conclusions

In this retrospective, single-center study, non-smoking status, CTCAE Grade ≤ 2, lower serum KL-6 levels at onset, low cumulative steroid dose, and short duration of steroid therapy were associated with CIP relapse. Relapse tended to occur in patients with relatively milder pneumonitis who received lower cumulative steroid doses and shorter durations of steroid therapy. However, given the small sample size and potential statistical limitations, these findings should be interpreted with caution. The results should be considered exploratory and hypothesis-generating. Further large-scale studies are needed to clarify the optimal steroid treatment strategy for preventing CIP relapse.

## Figures and Tables

**Table 1:** Analysis of patient characteristics and statistical comparisons between the relapse and no-relapse groups.

	Total (*n* = 39)	Relapse (*n* = 13)	No Relapse (*n* = 26)	*p*-Value	FDR *Q*-Value
Age (years)	72 (67, 77)	72 (66, 76)	72 (67.8, 77.5)	0.86	1.00
Male, *n* (%)	35 (89.7)	11 (84.6)	24 (92.3)	0.59	0.85
Smoking history, *n* (%)	34 (87.2)	9 (69.2)	25 (96.2)	**0.035**	0.14
Brinkman index	960 (375, 1380)	700 (0, 1720)	980 (544, 1350)	0.56	0.84
Cre (mg/dL)	0.85 (0.76, 1.01)	0.87 (0.79, 0.99)	0.83 (0.74, 1.01)	0.41	0.75
eGFR (mL/min/1.73 m^2^)	70.4 (56.0, 79.2)	66.2 (56.8, 75.9)	70.5 (56.7, 80.7)	0.58	0.85
Antinuclear antibody positive, *n* (%)	24 (61.5)	9 (69.2)	15 (57.7)	0.73	0.93
History of lung radiation exposure, *n* (%)	9 (23.0)	5 (38.5)	4 (15.4)	0.13	0.33
Presence of lung lesions, *n* (%)					
Primary or metastatic cancer lesions	31 (79.5)	11 (84.6)	20 (76.9)	0.69	0.92
Interstitial abnormalities	4 (10.2)	1 (7.7)	3 (11.5)	1.0	1.0
Emphysema	24 (61.5)	7 (53.8)	17 (65.4)	0.51	0.84
Old inflammatory changes	13 (33.3)	3 (23.0)	10 (37.0)	0.48	0.83
Primary cancer, *n* (%)					
Lung	28 (71.7)	9 (69.2)	19 (73.1)	1.0	1.0
Renal	3 (7.7)	1 (7.7)	2 (7.7)	1.0	1.0
Esophageal	3 (7.7)	2 (15.4)	1 (3.8)	0.25	0.50
Head and neck	3 (7.7)	1 (7.7)	2 (15.4)	0.54	0.84
Melanoma	2 (5.1)	0	2 (7.7)	1	1.0
Type of ICI, *n* (%)					
Pembrolizumab	23 (59.0)	5 (38.5)	18 (69.2)	0.091	0.27
Nivolumab	9 (23.1)	5 (38.5)	4 (15.4)	0.13	0.33
Durvalumab	4 (10.2)	3 (23.1)	1 (3.8)	0.099	0.30
Nivolumab and ipilimumab	2 (5.1)	0 (0)	2 (7.7)	0.54	0.84
Atezolizumab	1 (2.6)	0 (0)	1 (3.8)	1.0	1.0
Avelumab	0	0	0	-	
Ipilimumab only	0	0	0	-	
Number of ICIs administered	6.0 (3.0, 12.5)	6.0 (4.0, 12.0)	6.5 (3.0, 12.8)	0.88	1.0
Use of combined medicines, *n* (%)	8 (26)	0 (0)	6 (30.8)	0.081	1.0
Number of days from first ICI dose to CIP onset	124 (69, 271)	110 (65, 212)	180 (74, 305)	0.33	0.62
CTCAE grade, *n* (%)					
Grade 2	26 (66.7)	12 (92.3)	14 (53.8)	**0.029**	0.13
Grade 3 or higher	13 (33.3)	1 (7.7)	12 (46.2)		
LDH (U/L)	241 (186, 299)	207 (186, 279)	263 (196, 308)	0.32	0.62
CRP (mg/dL)	3.29 (0.76, 7.51)	3.61 (0.93, 8.44)	2.45 (0.46, 7.18)	0.36	0.66
KL-6 (U/mL)	691 (313, 856)	288 (268, 625)	704 (538, 1248)	**0.014**	0.11
Pattern of CT findings, *n* (%)					
OP	18 (46.1)	6 (46.2)	12 (46.2)	1.0	1.0
NSIP	8 (20.5)	5 (38.5)	3 (11.5)	0.090	1.0
DAD	5 (12.8)	0 (0)	5 (19.2)	0.15	0.68
HP	3 (7.7)	1 (7.7)	2 (7.7)	1.0	1.0
Bronchitis	1 (2.5)	0 (0)	1 (3.8)	1.0	1.0
Other	4 (10.3)	1 (7.7)	3 (11.5)	1.0	1.0
Cumulative steroid dose (mg)	1688 (1059, 3184)	1140 (700, 1688)	1902 (1155, 4531)	**0.015**	0.675
Starting steroid dose, *n* (%)					
PSL 0.5 mg/kg/day	23 (59.0)	9 (69.2)	14 (53.8)	0.50	1.0
PSL 1 mg/kg/day	6 (15.4)	2 (15.4)	4 (15.4)	1	1.0
Steroid pulse	10 (25.6)	2 (15.4)	8 (30.8)	0.45	1.0
Duration of steroid treatment					
Overall number of days with steroids	86 (63, 136)	63 (42, 106)	101 (74, 150)	**0.038**	1.0
Number of days with PSL starting dose	14 (28, 10))	7 (7, 10)	14 (8, 14)	**0.025**	1.0
Number of days with PSL ≥ 0.5 mg/kg/day	14 (10, 21)	10 (7, 14)	14 (14, 21)	**0.029**	1.0
Number of days with PSL ≥ 20 mg/day	29 (21, 42)	21 (14, 28)	35 (28, 56)	**0.0036**	0.162
Number of days with PSL ≥ 15 mg/day	42 (28, 58)	27 (22, 45)	46 (34, 63)	**0.013**	0.585

Data are expressed as median (IQR). Bold values indicate statistical significance (*p* < 0.05). Cre, creatinine; eGFR, estimated glomerular filtration rate; ICIs, immune checkpoint inhibitors; CIP, checkpoint-inhibitor-related pneumonitis; CTCAE, Common Terminology Criteria for Adverse Events; LDH, lactate dehydrogenase; CRP, C-reactive protein; KL-6, Krebs von den Lungen-6; CT, computed tomography; OP, organizing pneumonia; NSIP, non-specific interstitial pneumonia; DAD, diffuse alveolar damage; HP, hypersensitivity pneumonia; PSL, prednisolone.

**Table 2:** Characteristics of the 13 patients in the relapse group.

Age /Sex	Cancer	Drug	Smoking History	KL-6 (U/mL)	CT Findings	CTCAE Grade	Days From First ICI Dose to Relapse	Days From Last ICI Dose to Relapse	Days From Start of Steroid to Relapse	Starting Steroid Dose	PSL Dose at Relapse (mg)
36 M	Head and neck	Nivolumab	−	268	OP	2	338	35	24	PSL 0.5 mg/kg	15
61 M	Esophageal	Nivolumab	+	766	HP	2	319	83	70	PSL 0.5 mg/kg	0
76 M	Renal	Nivolumab	−	1527	OP	2	244	167	140	PSL 0.5 mg/kg	0
78 M	Esophageal	Nivolumab	+	245	NSIP	2	59	80	59	PSL 0.5 mg/kg	0
66 M	Lung	Nivolumab	+	186	other	2	188	91	78	mPSL pulse	5
71 F	Lung	Pembrolizumab	−	541	OP	2	247	171	157	mPSL pulse	1
66 M	Lung	Pembrolizumab	+	269	OP	2	330	98	59	PSL 1 mg/kg	7.5
72 F	Lung	Pembrolizumab	−	338	OP	2	198	94	74	PSL 0.5 mg/kg	5
76 M	Lung	Pembrolizumab	+	730	NSIP	3	318	127	106	PSL 0.5 mg/kg	5
83 M	Lung	Pembrolizumab	+	625	OP	2	128	183	140	PSL 0.5 mg/kg	0
76 M	Lung	Durvalumab	+	288	NSIP	2	80	40	38	PSL 0.5 mg/kg	10
69 M	Lung	Durvalumab	+	276	NSIP	2	231	156	140	PSL 0.5 mg/kg	2.5
79 M	Lung	Durvalumab	+	256	NSIP	2	84	73	63	PSL 0.5 mg/kg	17.5

“+” indicates a positive smoking history, and “−” indicates no smoking history. KL-6, Krebs von den Lungen-6; CT, computed tomography; CTCAE, Common Terminology Criteria for Adverse Events; OP, organizing pneumonia; NSIP, non-specific interstitial pneumonia; PSL, prednisolone; mPSL, methylprednisolone.

**Table 3:** Analysis of the 23 patients who completed steroid treatment.

	Total (*n* = 23)	Relapse (*n* = 4)	No Relapse (*n* = 19)	*p*-Value	FDR *Q*-Value
Number of days with PSL < 15 mg/day	42 (21, 77)	32 (20, 54)	56 (25, 84)	0.54	0.54
Number of days with PSL < 20 mg/day	63 (29, 91)	42 (32, 63)	63 (32, 100)	0.49	0.54
Overall number of days with steroids	84 (38, 126)	52 (41, 77)	86 (68, 143)	0.10	0.18
Number of days with PSL starting dose	13 (9, 14)	9 (7, 11)	14 (10, 15)	0.18	0.25
Number of days with PSL ≥ 0.5 mg/kg/day	14 (9, 21)	7 (7, 9)	14 (14, 21)	**0.031**	0.072
Number of days with PSL ≥ 20 mg/day	28 (21, 39)	14 (12, 16)	34 (26, 44)	**0.0064**	0.022
Number of days with PSL ≥ 15 mg/day	40 (28, 53)	21 (21, 23)	42 (35, 58)	**0.0056**	0.022

Data are expressed as median (IQR). Bold values indicate statistical significance (*p* < 0.05). PSL, prednisolone.

**Table 4:** Univariate analysis with CIP relapse as the outcome.

	HR	95% CI	*p*-Value
Patient characteristics			
Male	0.46	0.057–3.69	0.46
No smoking history	11.10	1.09–113.00	**0.042**
Antinuclear positive	1.65	0.042–6.77	0.49
History of lung radiation exposure	3.44	0.73–16.10	0.12
Presence of lung lesions			
Primary or metastatic cancer lesions	1.65	0.28–9.60	0.58
Interstitial abnormalities	0.64	0.060–6.82	0.71
Emphysema	0.62	0.16–2.40	0.49
Old inflammatory changes	0.48	0.11–2.18	0.34
Lung cancer	0.83	0.19–3.58	0.80
Types of ICIs			
Pembrolizumab	0.28	0.69–1.12	0.072
Nivolumab	3.44	0.73–16.10	0.18
Durvalumab	7.5	0.70–81.00	0.097
Patterns of onset			
CTCAE Grade 2	10.30	1.16–91.10	**0.036**
Pattern of CT findings			
OP	1.00	0.26–3.80	1.00
NSIP	4.79	0.93–24.80	0.061
Serum KL-6 levels at onset ≤ 338 U/mL	12.30	2.37–63.40	**0.0028**
Treatment strategies			
Starting dose of steroid ≥ PSL 1mg/kg	0.52	0.13–2.12	0.36
Administration of steroid pulse at the start of treatment	0.41	0.073–2.29	0.31
Cumulative steroid dose ≤ 1688 mg	5.33	1.17–24.20	**0.031**
Overall number of days of steroid treatment ≤ 78 days	4.25	1.02–17.70	**0.047**
Number of days with PSL starting dose ≤ 10 days	3.89	0.87–17.50	0.077
Number of days with PSL ≥ 0.5mg/kg/day ≤ 10 days	5.33	1.26–22.60	**0.023**
Number of days with PSL ≥ 20 mg/day ≤ 29 days	10.40	1.88–57.40	**0.0073**
Number of days with PSL ≥ 15 mg/day ≤ 28 days	8.80	1.88–41.20	**0.0058**

Bold values indicate statistical significance (*p* < 0.05). ICIs, immune checkpoint inhibitors; OP, organizing pneumonia; NSIP, non-specific interstitial pneumonia; KL-6, Krebs von den Lungen-6; CT, computed tomography; CTCAE, Common Terminology Criteria for Adverse Events; PSL, prednisolone; HR, hazard ratio; 95% CI, 95% confidence interval.

**Table 5:** Multivariate analysis with CIP relapse as the outcome.

	HR	95% CI	*p*-Value
No smoking history	9.63	0.69–134.00	0.092
CTCAE Grade 2	6.99	0.57–85.20	0.13
Serum KL-6 levels at the onset ≤ 338 U/mL	14.90	2.14–104.00	**0.0063**

Bold values indicate statistical significance (*p* < 0.05). KL-6, Krebs von den Lungen-6; CTCAE, Common Terminology Criteria for Adverse Events; HR, hazard ratio; 95% CI, 95% confidence interval.

## Data Availability

The data presented in this study are available upon request from the corresponding author.

## Abbreviations

The following abbreviations are used in this manuscript:

ATS	American Thoracic Society
AUC	Area under the curve
CI	Confidence interval
CIP	Checkpoint-inhibitor-related pneumonitis
CT	Computed tomography
CTCAE	Common Terminology Criteria for Adverse Events
CTLA-4	Cytotoxic T-Lymphocyte-Associated protein-4
DAD	Diffuse alveolar damage
ERS	European Respiratory Society
HP	Hypersensitivity pneumonia
HR	Hazard ratio
KL-6	Krebs von den Lungen-6
ICIs	Immune checkpoint inhibitors
IQR	Interquartile range
mPSL	Methylprednisolone
NCCN	National Comprehensive Cancer Network
NSIP	Non-specific interstitial pneumonia
OP	Organizing pneumonia
PD-1	Programmed cell Death-1
PD-L1	Programmed cell Death Ligand 1
PS	Performance status
PSL	Prednisolone
ROS	Receiver operating characteristic
